# DFT study on tunable electronic and adsorption properties of poly(vinyl alcohol)/copper oxide/graphene oxide hybrid nanostructures

**DOI:** 10.1038/s41598-026-54159-y

**Published:** 2026-05-26

**Authors:** Asmaa Ibrahim, Mervat Abd El Aal, Hayam El-Zahed, Ahmed F. Mabied

**Affiliations:** 1 Physics Department, Faculty of Women for Arts, Science and Education, Ain Shams University, 11757 Cairo, Egypt; 2 X-ray Crystallography Lab, Solid State Physics Department, National Research Center, 33 Bohouth St, Dokki, 12622 Egypt; 3 Physics Department, Faculty of Science, Galala University, New Galala City, 43511 Suez Egypt

**Keywords:** PVA/CuO/GO, DFT, Electronic properties, HOMO-LUMO energy gap, QTAIM, Global reactivity, Adsorption energy, Chemistry, Materials science, Nanoscience and technology

## Abstract

The rapid development of nanoelectronics and environmental monitoring requires multifunctional polymeric materials with tailored electronic properties. In this work, Density Functional Theory (DFT) calculations at the B3LYP/LanL2DZ level are used to investigate the structural and electronic properties of poly(vinyl alcohol) (PVA) nanocomposites incorporating copper oxide (CuO) and graphene oxide (GO). The incorporation of CuO and GO significantly reduces the energy gap (ΔE) from 7.334 eV in pristine PVA to 1.415 eV for the Cu-mediated PVA–Cu/CuO/GO model and further to 0.819 eV for the oxygen-mediated PVA–O/CuO/GO configuration, indicating enhanced semiconducting behavior. Molecular electrostatic potential (MESP), density of states (DOS), and frontier orbital analyses reveal charge redistribution and the formation of interfacial states near the Fermi level. Non-covalent interaction (NCI), reduced density gradient (RDG), and QTAIM analyses confirm extensive hydrogen bonding and dispersive interactions across the interfaces. Gas adsorption studies show that H₂O and CO₂ adsorption increase the total dipole moment (TDM) and modulate ΔE, as seen in PVA–Cu/CuO/GO–2 H₂O (TDM = 10.447 Debye, ΔE = 1.112 eV) and PVA–O/CuO/GO–2CO₂ (TDM = 11.599 Debye, ΔE = 2.446 eV). The adsorption energy for CO₂ on PVA–Cu/CuO/GO is − 0.406 eV, indicating favorable and reversible physisorption with partial charge transfer. Overall, the calculations demonstrated that the combination of PVA, CuO, and GO effectively tunes the electronic structure and enhances interfacial interactions, leading to improved sensitivity and selectivity for gas and humidity sensing applications in future research.

## Introduction

Polymer nanocomposites have emerged as an important class of hybrid materials due to their ability to combine the flexibility and processability of polymers with the unique electronic, optical, and surface properties of nanomaterials, enabling tunable physicochemical behavior for advanced functional applications such as sensing and electronics^1–3^. Among various polymers, polyvinyl alcohol (PVA) stands out because of its film-forming ability, chemical stability, non-toxicity, and high density of hydroxyl groups that promote strong interfacial interactions with nanofillers^4–6^. However, pristine PVA exhibits a wide electronic energy gap and low intrinsic conductivity, which limits its direct use in electronic and sensing devices^7^. To enhance these properties, integrating inorganic nanofillers such as metal oxides and carbon-based materials has been widely explored^8–10^.

Copper oxide (CuO) is a p-type semiconducting metal oxide with a relatively narrow energy gap, a rich surface chemistry, and strong interaction potential with adsorbed molecules. At the nanoscale, CuO facilitates charge transfer and contributes localized electronic states that can significantly modify the electronic structure of host matrices^11–13^. Graphene oxide (GO), a derivative of graphene functionalized with oxygen-containing groups (e.g., hydroxyl, epoxy, carboxyl), demonstrates a large surface area, enhanced dispersibility in polymer matrices, and significant charge delocalization characteristics, making it an effective nanofiller for tuning polymer electronic properties^14–17^. The combination of CuO and GO within PVA results in a ternary nanocomposite with synergistic enhancements in structural stability and electronic responsiveness. CuO introduces active semiconducting centers that can lower the energy gap and promote charge transfer, while GO contributes extended π-electron networks and facilitates charge delocalization across the composite^18–20^. Despite experimental progress on PVA–metal oxide–GO systems, a detailed theoretical understanding of their electronic structure and interfacial interactions remains essential to guide rational design for specific applications.

A strong foundation for examining the electrical structure, charge transfer, and reactivity of intricate nanostructured systems is provided by Density Functional Theory (DFT). It enhances experimental research by enabling precise prediction of molecule characteristics, electronic dispersion, and intermolecular interactions^21,22^. Density Functional Theory (DFT) has been successfully applied to investigate the fundamental electronic properties of polymer nanocomposites.

The calculation of frontier molecular orbitals (HOMO and LUMO), energy gap (∆E), dipole moment, density of states (DOS), and molecular electrostatic potential (MESP) offers comprehensive insight into structural stability, charge distribution, and reactivity of complex systems^23–26^. Previous theoretical studies have shown that incorporation of GO and metal oxides into polymer matrices significantly lowers ∆E and enhances charge polarization, consistent with improved functional performance^27,28^. Moreover, adsorption studies using DFT have demonstrated that nanocomposite surfaces interact preferentially with gas molecules such as CO₂ and H₂O through electrostatic and hydrogen-bonding mechanisms, affecting sensitivity and selectivity in sensing applications^29–31^. This study presents a novel theoretical investigation of PVA/CuO/GO nanocomposites through DFT, utilizing advanced analyses (HOMO–LUMO, DOS, MESP, QTAIM, and NCI) to understand how the electronic structure influences adsorption of H₂O and CO₂ molecules, preparing the field for future studies aimed at enhancing these nanocomposites for advanced gas and humidity detection applications. Moreover, this study illustrates the usefulness of computational methods in polymer research and corresponds with earlier frameworks, like antioxidant screening in cross-linked polyethylene, emphasizing their promise in directing the design of advanced functional materials^32^. Because of its demonstrated dependability for comparable nanostructured systems, the B3LYP functional was used^33^. The LanL2DZ basis set was used for all atoms; although it was created for heavier elements, it has been effectively used for light atoms in earlier research, guaranteeing a balance between computing cost and precision^34^.

This study systematically investigates PVA/CuO/GO nanocomposites using Density Functional Theory (DFT) at the B3LYP/LanL2DZ level. Special attention is given to the modulation of the HOMO-LUMO energy gap, charge redistribution, dipole moment, and non-covalent interactions. To elucidate how the synergistic integration of CuO and GO alters the electronic structure and interaction energetics, theoretical descriptors including HOMO-LUMO analysis, Density of States (DOS), Molecular Electrostatic Potential (MESP), Quantum Theory of Atoms in Molecules (QTAIM), and Non-Covalent Interaction (NCI) analysis are employed. Furthermore, the nanocomposite interactions with H₂O and CO₂ molecules are examined to evaluate its adsorption behavior and molecular-level reactivity. These findings provide a robust foundation for understanding and optimizing the design of polymer nanocomposites with tailored electronic and adsorption characteristics for advanced functional applications.

## Computational details

All quantum chemistry calculations for the PVA/CuO/GO structures were conducted using the Gaussian 09 software package^35^, employing the B3LYP functional and the LanL2DZ basis set^36–38^. The optimized structures were confirmed as minima through vibrational frequency analysis conducted after geometry optimizations without symmetry constraints. Infrared (IR) spectra, energy gap (ΔE), molecular electrostatic potential (MESP), total dipole moment (TDM), density of states (DOS) and projected density of states (PDOS), as well as additional electronic and structural properties were assessed. In order to gain a deeper insight into the interactions occurring within composite systems, the stability of the model structures under examination was further explored through Quantum Theory of Atoms in Molecules (QTAIM) analysis using Multiwfn and VMD software^39,40^. Employing reduced density gradient (RDG) research, noncovalent interactions (NCI) were examined to demonstrate weak intermolecular forces like hydrogen bonds and van der Waals contacts. Moreover, to evaluate the overall chemical reactivity and stability of the complexes being studied, global reactivity descriptors such as ionization potential (I), electron affinity (A), chemical potential (µ), hardness (η), softness (S), and electrophilicity index (ω) were computed using the following equations^41^.

$${\mathrm{I}} = {\text{ }} - {\mathrm{E}}_{{{\mathrm{HOMO}}}}$$

$${\mathrm{A}} = {\text{ }} - {\mathrm{E}}_{{{\mathrm{LUMO}}}}$$

$$\mu = {\text{ }} - \left( {{\mathrm{I}} + {\mathrm{A}}} \right)/{\mathrm{2}}$$

$$\eta = {\text{ }}\left( {{\mathrm{I}} - {\mathrm{A}}} \right)/{\mathrm{2}}$$

$${\mathrm{S}} = {\text{ 1}}/\eta$$

$$\omega = {\text{ }}\mu ^{{\mathrm{2}}} /{\mathrm{2}}\eta$$

## Results and discussion

### Model building

**Fig. 1:**
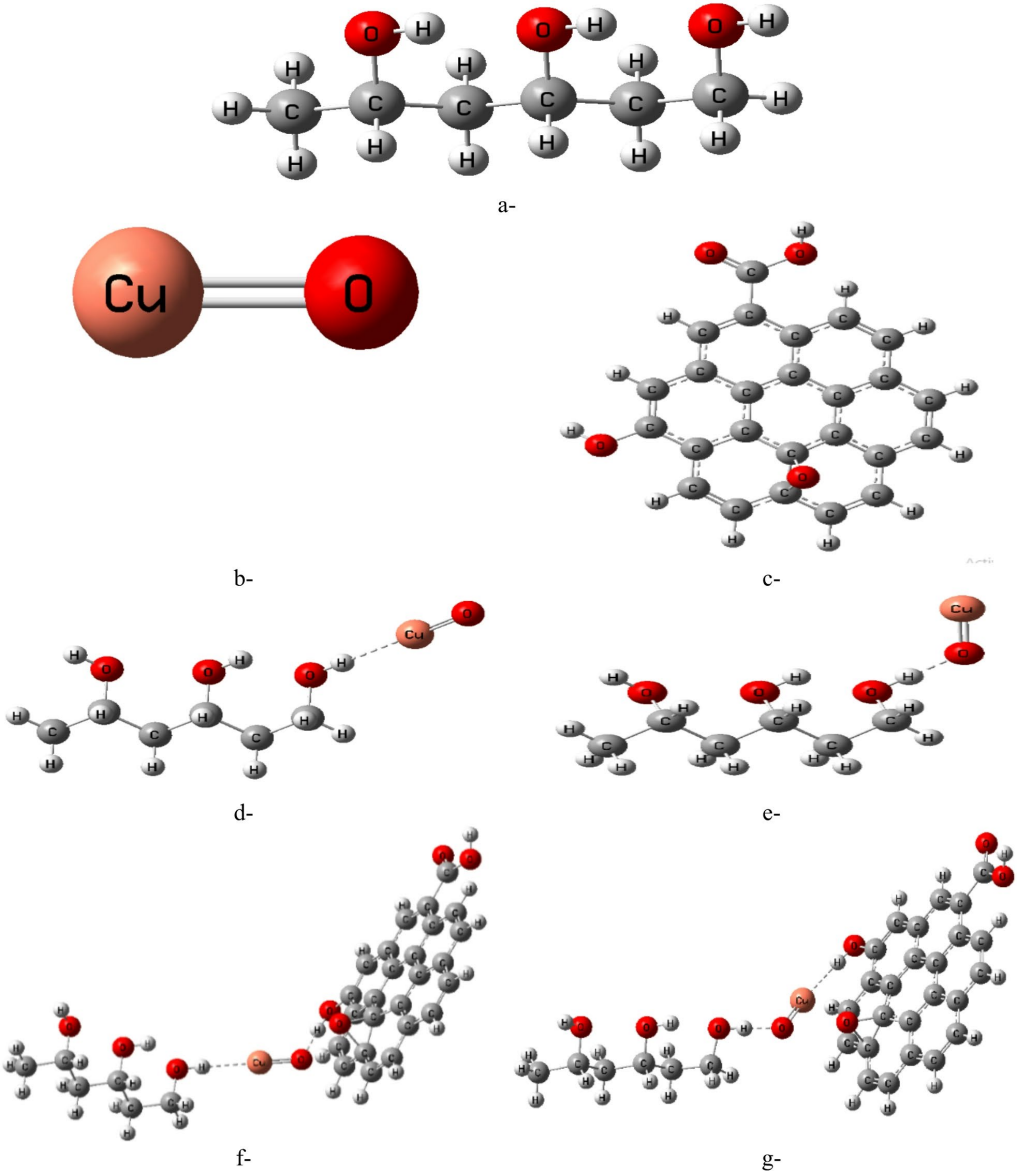
Structures of optimized models for: (**a**) PVA, (**b**) CuO, (**c**) Graphene oxide (GO), (**d**) PVA-Cu/CuO, e-PVA-O/CuO, f-PVA-Cu/CuO/GO and g-PVA-O/CuO/GO.

Three repeating units of PVA were used to construct the molecular model, as shown in Fig. 1-a, providing a reasonable representation of polymer nanocomposite interactions^42^. Figure 1-b illustrates the CuO model, which interacts with the hydroxyl (OH) groups of PVA through interfacial bonding mechanisms involving both the copper and oxygen surface sites of CuO. The Cu-mediated interaction involves the formation of Cu–O–C coordination bonds between CuO and the hydroxyl groups of PVA, whereas the oxygen-mediated interaction mainly originates from interfacial hydrogen bonding of the type O–H···O–Cu. These interactions, denoted as PVA–Cu/CuO and PVA–O/CuO, are presented in Fig. 1d and e, respectively.

The incorporation of graphene oxide (GO), shown in Fig. 1-c, was motivated by its ability to introduce additional weak interfacial interactions within the PVA/CuO network through its oxygen-containing functional groups. In the ternary PVA/CuO/GO system, both the Cu and O atoms of CuO contribute to interfacial interactions with the hydroxyl groups of PVA and the functional groups of GO, as illustrated in Fig. 1f and g, respectively. The existence of these interactions enhances the formation of a synergistic hybrid structure characterized by Cu-mediated coordination, oxygen-mediated hydrogen bonding, and GO-assisted interfacial stabilization within the nanocomposite matrix. Accordingly, PVA–Cu/CuO/GO denotes Cu-mediated interfacial coordination, whereas PVA–O/CuO/GO represents oxygen-mediated interaction between PVA and CuO.

**Fig. 2:**
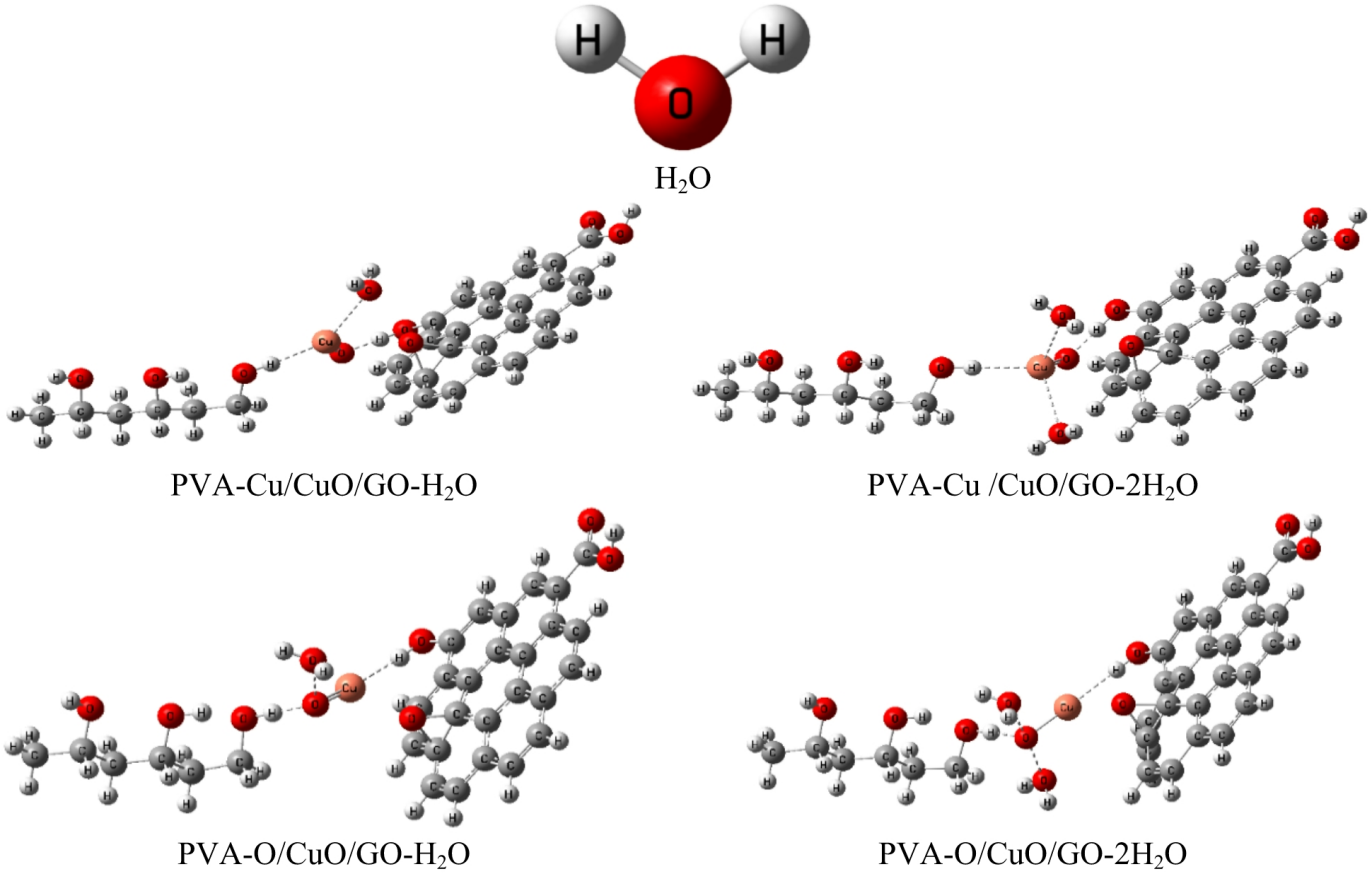
Structures of optimized models for the interaction of PVA-Cu/CuO/GO and PVA-O/CuO/GO with H_2_O and 2H_2_O.

**Fig. 3:**
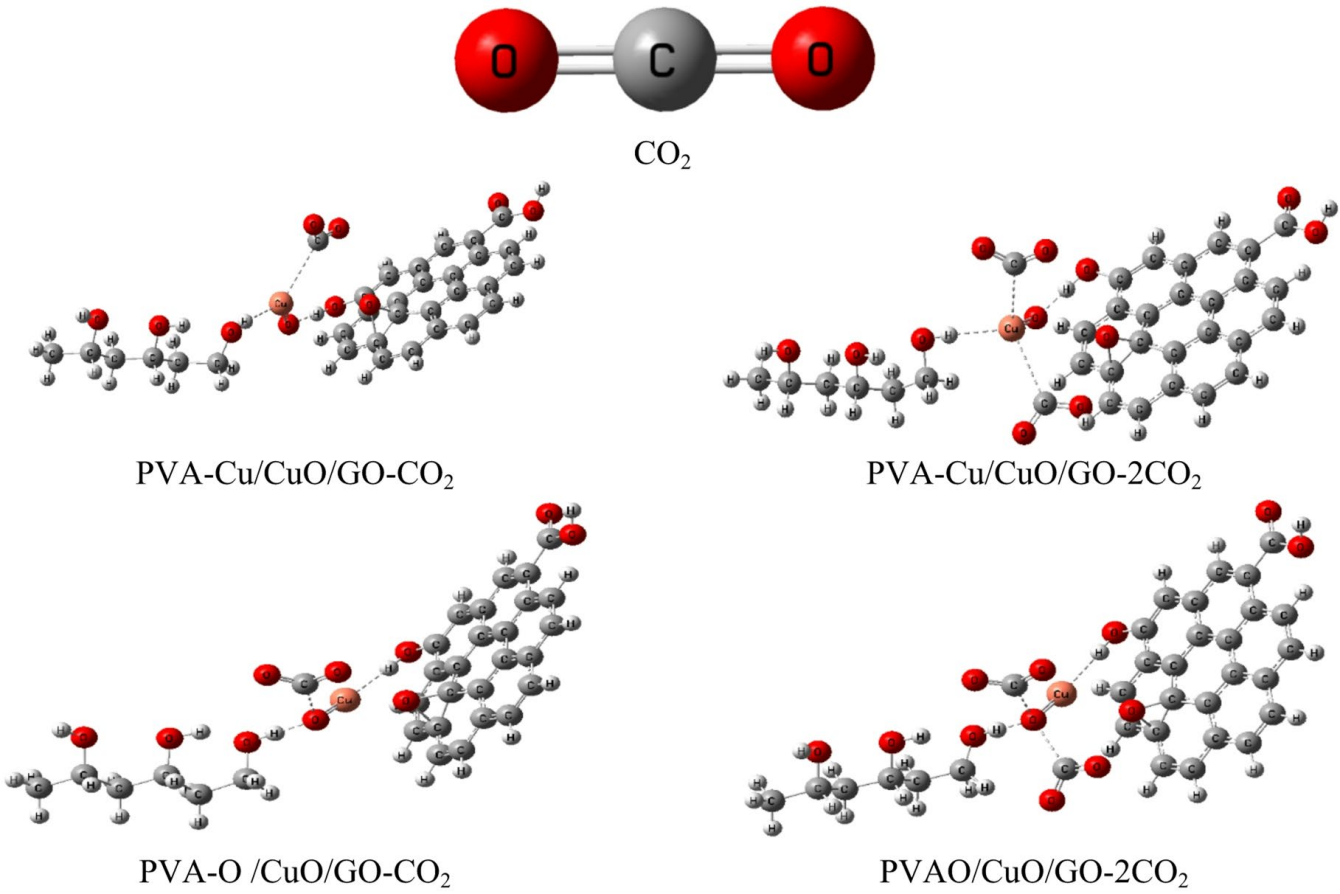
Structures of optimized models for the interaction of PVA-Cu/CuO/GO and PVA-O/CuO/GO with CO_2_ and 2CO_2_.

The optimized molecular structures that show the PVA/CuO/GO composite interacts weakly with one or two molecules of water (H₂O) and carbon dioxide (CO₂) as shown in Figs. 2 and 3, respectively. For both the PVA-Cu/CuO/GO and PVA–O/CuO/GO structures, the weak contact was modeled from two angles: once through the Cu atom and once through the oxygen atom of CuO. The purpose of this research is to examine how sensitive the composites are to exposure to gases and humidity. GaussView 5.0 software was used to create each model molecule^43^.

### Calculated IR for CuO, GO and PVA/CuO/GO composites

#### Calculated IR for CuO and GO

**Fig. 4:**
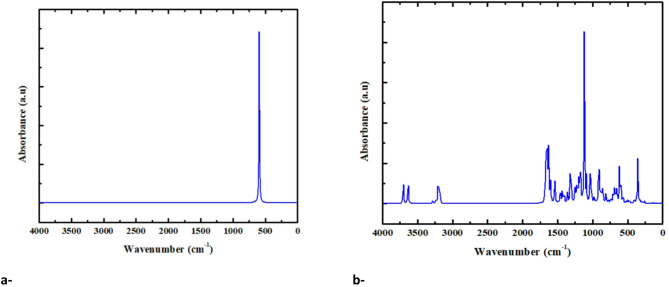
Calculated IR absorbance spectra (B3LYP/LanL2DZ) for, a- CuO and b- Graphene oxide (GO).

Theoretical calculations using the B3LYP/LanL2DZ method were performed to obtain the calculated IR spectra of CuO and GO, as shown in Fig. 4. In Fig. 4-a, a prominent absorption peak at 591 cm⁻¹ is observed, resulting from Cu–O stretching. Absorption bands at 3704, 3640, 3208, 1664, 1632, 1320, 1120, 1042, 912, 864, 616, and 360 cm⁻¹ are shown in the GO spectrum (Fig. 4-b). The bands are ascribed to the stretching vibrations of O–H (3704–3640 cm⁻¹), O-H hydrogen bond stretching (3208 cm⁻¹), C = O (1664 cm⁻¹), C = C (1632 cm⁻¹), and C–O bonds (360–1320 cm⁻¹), signifying the existence of different oxygen-containing functional groups on the GO surface^44–46^. The calculated spectra for both CuO and GO demonstrate the reliability of the DFT-based B3LYP/LanL2DZ method in predicting vibrational features and provide strong evidence for the structural models used.

#### Calculated IR for PVA/CuO/GO composites

**Fig. 5:**
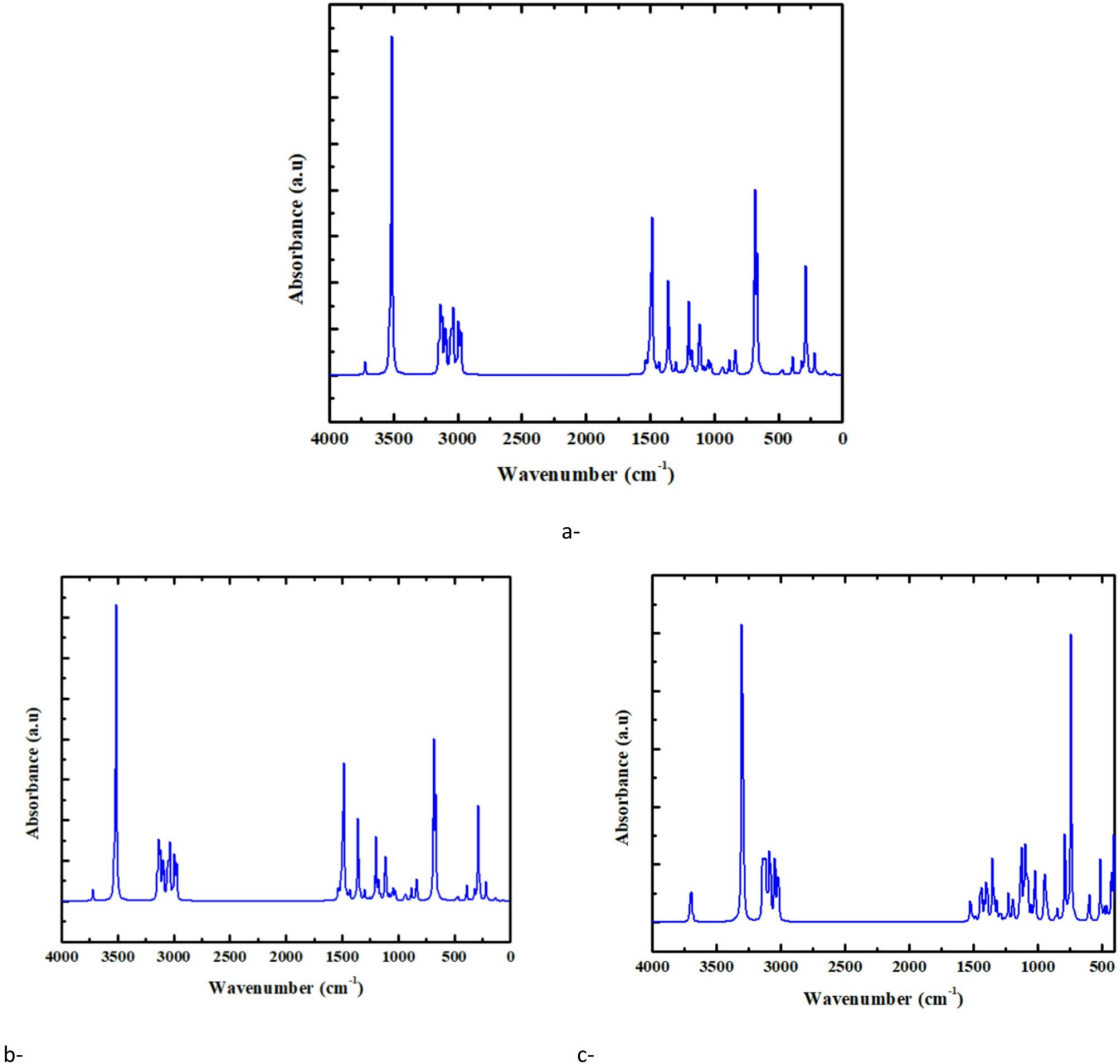

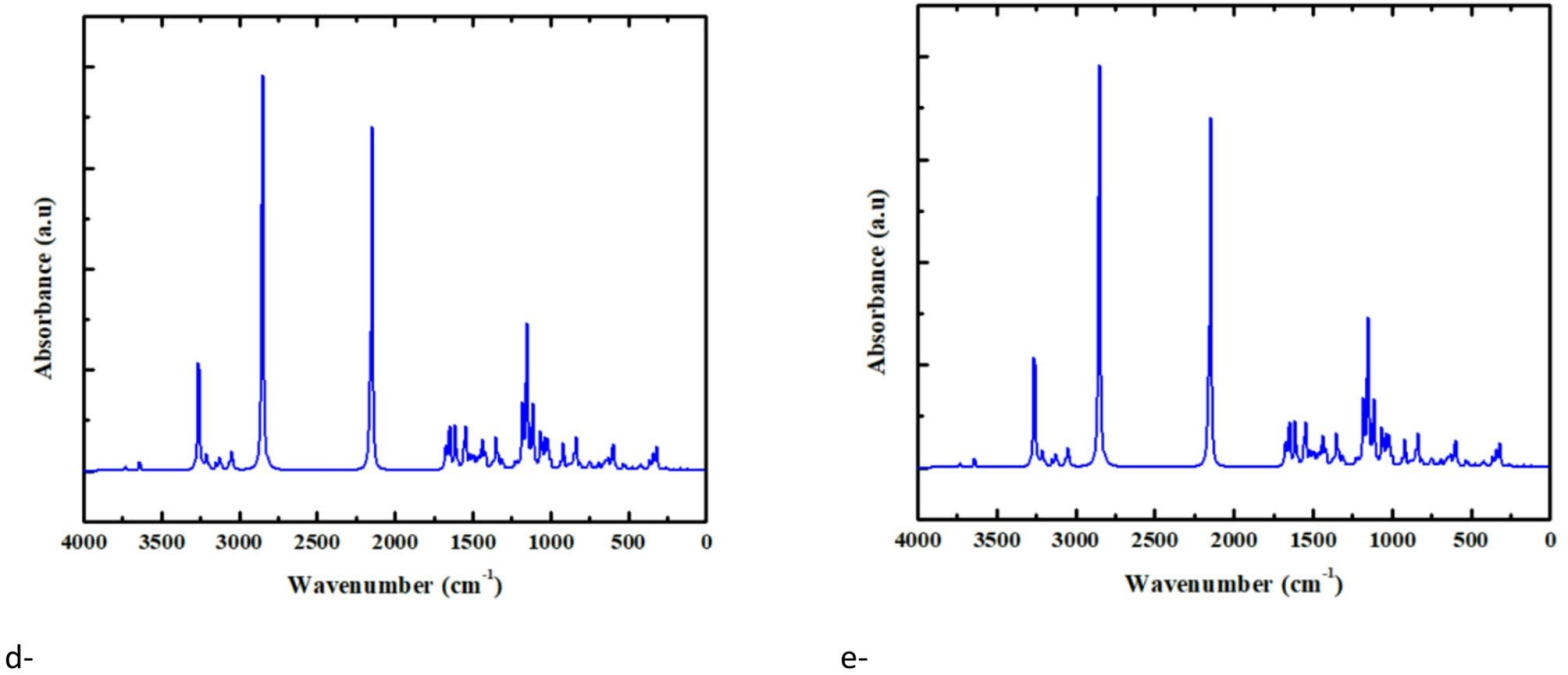
Calculated IR absorbance spectra (B3LYP/LanL2DZ) for: (**a**) PVA, (**b**) PVA-Cu/CuO, (**c**) PVAO/CuO, (**d**) PVA-Cu/CuO/GO and (**e**) PVA-O/CuO/GO. The absence of imaginary frequencies in all spectra verifies that the optimized geometries correspond to genuine local energy minima and are dynamically stable.

Calculated IR spectra of PVA and its composite mixtures containing CuO and GO are illustrated in Fig. 5. The spectra show typical absorption bands for PVA between 3300 and 3500 cm⁻¹, linked to O-H stretching vibrations, which signifies strong hydrogen bonding in the polymer matrix. The extra band seen at around 1000–1300 cm⁻¹ corresponds to C-O stretching and C-H bending modes. When PVA is combined with CuO (Fig. 5b and c) and CuO/GO (Fig. 5d and e), significant changes occur, including increased intensity and slight shifts in the O-H and C-O stretching bands. This indicates strong interactions between the polymer matrix and the nanocomposite. Prominent bands linked to O-H, C-H, and C-O stretching modes are noted, with minor differences in wavenumber ascribed to computational approximations.

It can be seen clearly in Figs. 4 and 5 that the calculated spectra do not exhibit any negative values. The lack of negative values suggests that the optimized structure aligns with a genuine energy minimum on the potential energy surface. Consequently, the examined compound is structurally stable and does not signify a transition state.

### TDM and HOMO/LUMO energy gap

The intensity of electronic transitions is indicated by TDM values, with greater values signifying more effective electron excitation. HOMO-LUMO gap refers to the energy difference between the highest occupied molecular orbital and the lowest unoccupied molecular orbital, influencing the material’s electronic and optical properties. The computed TDM values and HOMO-LUMO energy gaps for the structures under investigation are shown in Table 1. The values presented in the table demonstrate significant variation among the structures, with the energy gap decreasing from 7.334 eV for PVA to 1.415 eV when PVA interacts with CuO/GO. The composite of PVA-O/CuO/GO exhibits the smallest energy gap of 0.819 eV, indicating a greater ease of electronic excitation, as well as a high TDM value of 11.621 Debye. The combination of a high TDM and a low energy gap suggests that PVA-O/CuO/GO has better electronic properties than the other structures. Its heightened reactivity makes the PVA/CuO/GO composite especially appropriate for application as a gas or humidity sensor.

**Table 1:** Dipole moments (TDM, Debye) and HOMO-LUMO energy gaps (∆E, eV) for the studied structures, calculated at the B3LYP/LanL2DZ level. The outcomes provide a quantification of the changes in electronic reactivity and molecular polarity that occur with the formation of nanocomposites.

Structural	TDM (Debye)	ΔE (eV)
PVA	7.148	7.334
CuO	4.651	3.137
GO	2.373	3.373
PVA/CuO	13.42	3.123
PVA/OCu	7.486	3.105
PVA/CuO/GO	10.960	1.415
PVA/OCu/GO	11.621	0.819

### Molecular electrostatic potential (MESP) maps

**Fig. 6:**
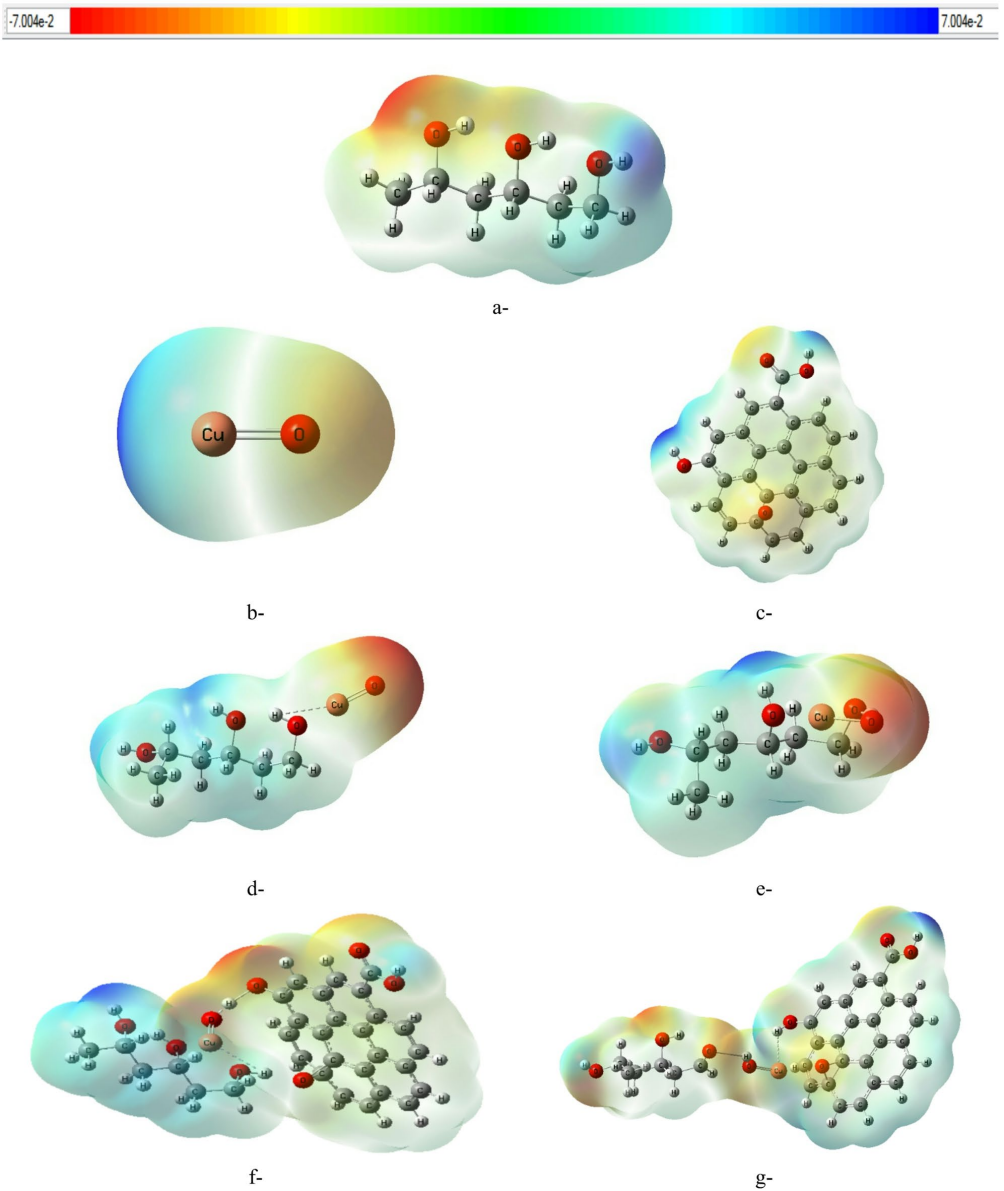
MESP maps determined at the B3LYP/LanL2DZ level for, (**a**) PVA, (**b**) CuO, (**c**) Graphene oxide (GO), (**d**) PVA-Cu/CuO, (**e**) PVA-O/CuO, (**f**) PVA-Cu/CuO/GO and (**g**) PVA-O/CuO/GO. The electrostatic potential distribution is illustrated by the color scale, which emphasizes the nucleophilic (red) and electrophilic (blue) areas that participate in the interaction.

MESP maps facilitate a better understanding of how charge is distributed within molecules and materials. The color gradient applied in the MESP maps adheres to a scientific standard, transitioning from red (nucleophilic sites, areas of high electron density) to yellow/green (moderate potential) and ultimately blue (electrophilic sites, areas of low electron density). understanding these maps is necessary for comprehension of reactivity, stability, and molecular interactions. Figure 6 displays the MESP calculations for PVA, CuO, and GO, as well as their composites formed through interactions involving either copper or oxygen atoms.

The findings indicate that the O-atom of CuO and the hydroxyl (OH) groups of the composites possess the most beneficial distribution of charge as shown in Fig. 6. The MESP distribution of the PVA-O/CuO/GO (Fig. 6-g) system is superior to that of the other tested composites, indicating enhanced stability (Fig. 6) and more robust intermolecular interactions. The investigation of HOMO-LUMO energies provides additional backing for these findings, revealing that enhanced electronic features are manifested in a charge distribution improvement that corresponds with a suitable energy gap. The results confirm the connection between charge distribution and electrical behavior in the studied composites, aligning well with the pervious studied and the data presented in Table 1[9, 23].

### Density of states calculations

**Fig. 7:**
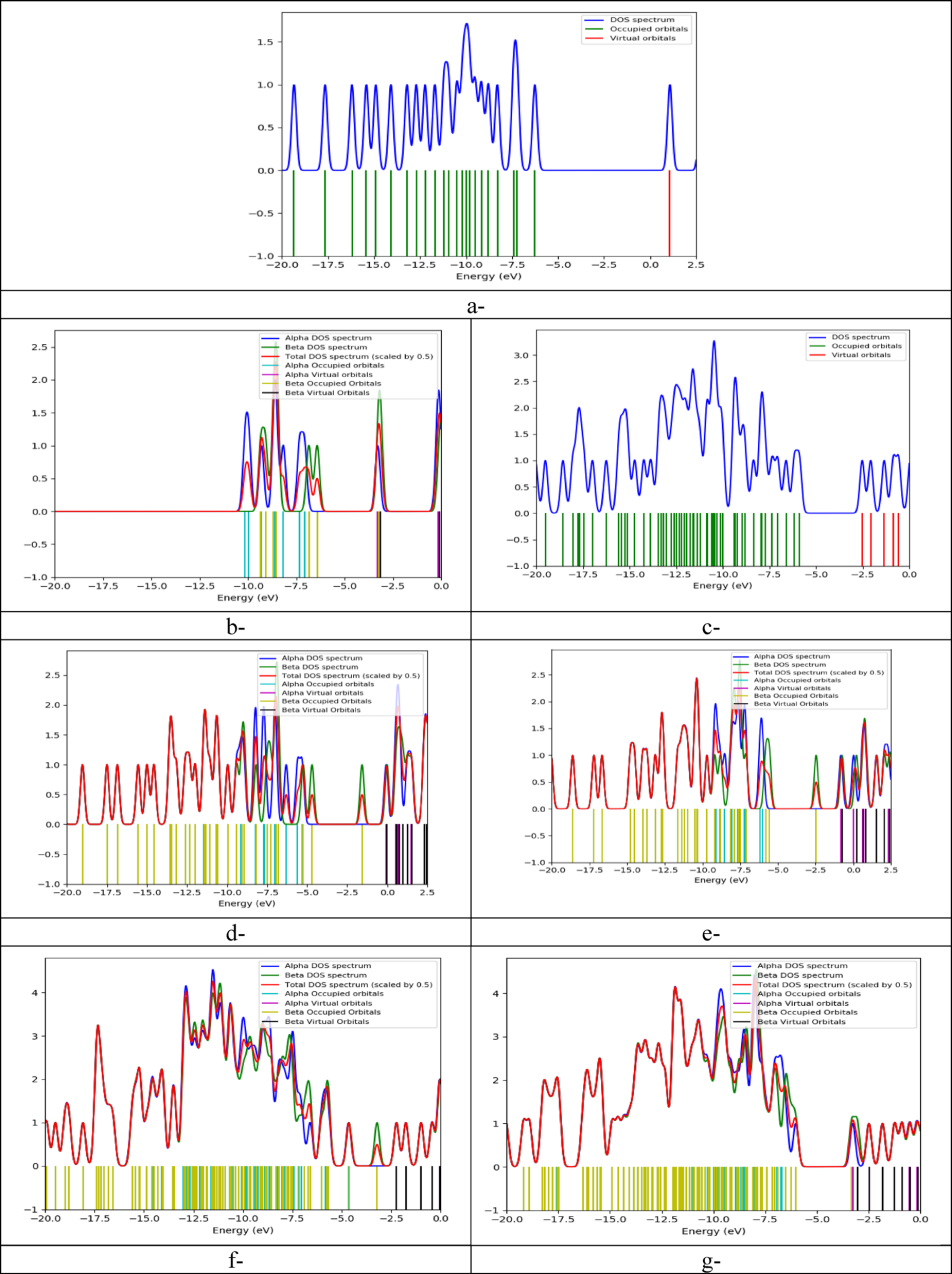
Density of States (DOS) calculated at the B3LYP/LanL2DZ level for (**a**) PVA, (**b**) CuO, (**c**) GO, (**d**) PVA-Cu/CuO, (**e**) PVA-O/CuO, (**f**) PVA-Cu/CuO/GO and (**g**) PVA-O/CuO/GO. The diagrams depict how the electronic energy levels are distributed and how the bandgap changes during the formation of a nanocomposite.

The DOS indicates how many electronic states at each energy level are accessible. Molecular orbitals that are occupied are represented by green lines in the figure, while those that are vacant (virtual) are indicated by red lines. By showing the energy difference between the highest occupied molecular orbital (HOMO) and the lowest unoccupied molecular orbital (LUMO), the DOS plot illustrates the chemical reactivity and electronic properties of the structures. A smaller HOMO-LUMO energy gap often indicates better electrical conductivity and increased chemical reactivity, while a larger energy gap suggests greater stability and reduced reactivity. Figure 7 shows the DOS for the examined structures, calculated using DFT at the B3LYP/LanL2DZ level. The DOS analysis of pure PVA, illustrated in Fig. 7-a, aligns with the theoretical calculations presented in Table 1 and reveals clear full and empty energy levels separated by a wide energy gap. The considerable energy gap indicates that pure PVA demonstrates effective insulating properties. Figures 7b and c illustrate the DOS for CuO and GO, respectively. The observable reduction in the energy gap resulting from the incorporation of CuO nanoparticles into the PVA matrix is illustrated in Fig. 7d and e. This can be accounted for by the interaction between the polymer chains and the CuO surface states, leading to the formation of localized energy levels in the energy gap. This interaction reduces the energy required for electronic transitions and enhances charge carrier movement. The addition of graphene oxide (GO) further modifies the electronic structures of the PVA/CuO composite, as shown in Fig. 7f and g. GO, with its extensive π-conjugated system and oxygen-containing functional groups, enhances interfacial contact and serves as an efficient charge transfer medium. This arrangement boosts charge delocalization and considerably narrows the energy gap by improving the overlap of the electronic states of PVA and CuO. When CuO is linked to the polymer through the oxygen atom (see Fig. 7-g), the energy gap decreases significantly, highlighting how crucial the orientation and bonding configuration of CuO are for adjusting the electronic structure of the composite. This arrangement is credited with enhancing orbital hybridization and interfacial charge transfer, which is said to fine-tune the energy gap. Previous first-principles studies of nanoscale materials^47^ have reported similar observations about the impact of orbital hybridization on electronic properties and adsorption behavior. The present DOS results agree well with the previous study of investigated PVA–GO–CuO nanocomposites using DFT, optical, and conductivity analyses^9^. Their results showed that CuO and GO incorporation introduces localized electronic states and enhances orbital overlap, leading to band-gap narrowing and improved charge-transfer behavior, consistent with the enhanced electronic delocalization observed in the present DOS calculations.

### Quantum theory of atoms in molecules (QTAIM) topology

**Fig. 8:**
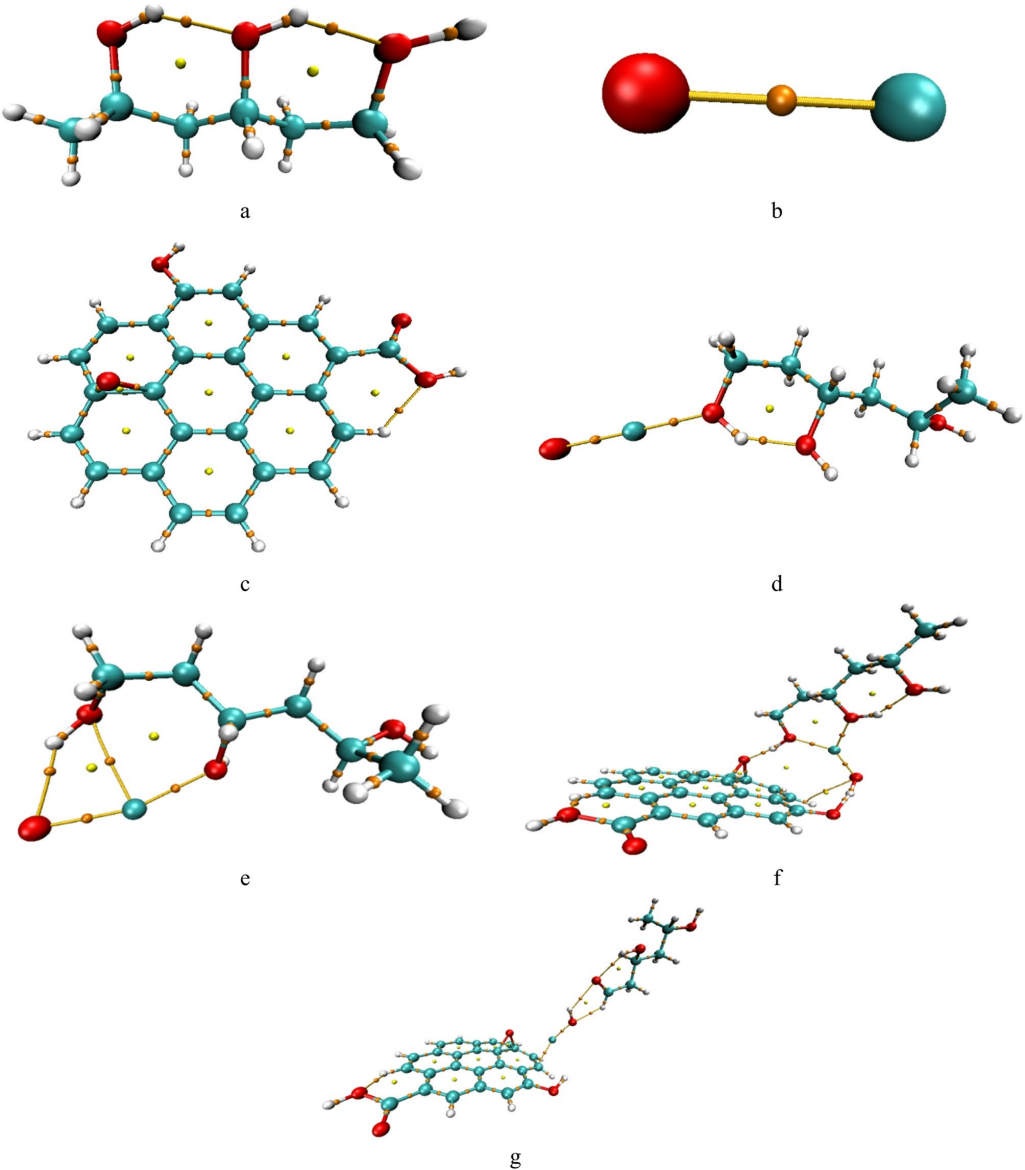
QTAIM molecular graphs showing bond paths and critical points for: (**a**) PVA, (**b**) CuO, (**c**) GO, (**d**) PVA-Cu/CuO, (**e**) PVA-O/Cu, (**f**) PVA-Cu/CuO/GO, and (**g**) PVA-O/Cu/GO. Bond Critical Points (BCPs) that control the interfacial stability are represented by small spheres.

Recent DFT investigations employing QTAIM analysis have demonstrated the critical role of interfacial charge redistribution and weak interactions in determining gas-sensing performance^48^. In order to clarify the nature of the bonding forces within the PVA/CuO/GO nanocomposite, a QTAIM topological analysis was conducted. The resulting interfacial descriptors and bond critical point (BCP) parameters, which fundamentally determine the physicochemical stability of the system, are summarized in Fig. 8; Table 2. In the case of the pure PVA system (Fig. 8-a), the QTAIM maps reveal characteristic bond critical points (BCPs) for covalent C-C, C-O, and O-H bonds, whereas the intermolecular regions exhibit low electron density, signifying minimal noncovalent interactions inherent to the polymer matrix. For isolated CuO (Fig. 8-b), the elevated electron density at the Cu-O BCP and the corresponding positive Laplacian values verify that the metal–oxygen bond has a partially ionic nature. Conversely, the GO structure (Fig. 8-c) shows many rings critical points (RCPs) and BCPs due to its vast conjugated framework. Oxygenated functional groups create localized electron density gradients that promote interfacial bonding with polymers. Upon the formation of the PVA-Cu/CuO composite (as shown in Fig. 8-d), new BCPs emerge between Cu/O sites and PVA’s hydroxyl groups, indicating the creation of robust hydrogen bonds and coordination interactions. In the PVA/GO composite (Fig. 8-e), multiple bond paths illustrate a synergistic effect of hydrogen bonding and π-interaction between the polymer and GO layers. This aligns with recent research indicating that efficient dispersion and robust bonding between matrix and filler enhance interfacial interaction in PVA/GO systems.

Significantly, the ternary PVA/CuO/GO hybrid (Fig. 8f and g) demonstrates the most intricate network of BCPs and bond paths, signifying an interconnected electron density distribution that encompasses PVA chains, CuO nanoparticles, and GO sheets. This topology, which is characterized by a high degree of interconnection, implies strong interfacial coupling that exhibits both covalent–ionic and hydrogen-bonded traits. Quantum topological analyses link this to increased charge transfer and interaction strength on the atomic scale. The presence of BCPs with ∇²ρ > 0 and H(r) < 0 for the Cu-O contacts confirms the formation of a coordination interaction with significant partially covalent character, which is responsible for the enhanced interfacial adhesion in the PVA/CuO/GO nanocomposite. These findings align with recent developments in QTAIM methodologies that stress the significance of a detailed electron density topology for comprehending the interfacial properties of materials and forecasting their functional performance^49,50^.

**Table 2:** Topological properties of the electron density at the Bond Critical Points (BCPs) for the key interactions in PVA/CuO/GO: Parameters include electron density (ρ), Laplacian of electron density (∇²ρ), and total energy density (H), utilized to characterize the nature of bonding (Covalent, Hydrogen bonding, and partially covalent) within the nanocomposite.

Bond	ρ(*r*) (a.u.)	∇²ρ(*r*) (a.u.)	H(*r*) (a.u.)	Interaction Character
O-H (PVA)	0.355	−1.121	−0.344	Shared-shell (Covalent)
Cu-O (CuO-PVA)	0.085	0.463	−0.015	Partially covalent (Coordination)
H···O (PVA-GO)	0.026	0.099	0.002	Hydrogen Bond (Electrostatic)
H···O (PVA-GO)	0.021	0.084	0.003	Hydrogen Bond
C···O (GO-PVA)	0.008	0.025	0.001	van der Waals (Weak)

### Non-covalent interaction (NCI) and reduced density gradient (RDG)

**Fig. 9:**
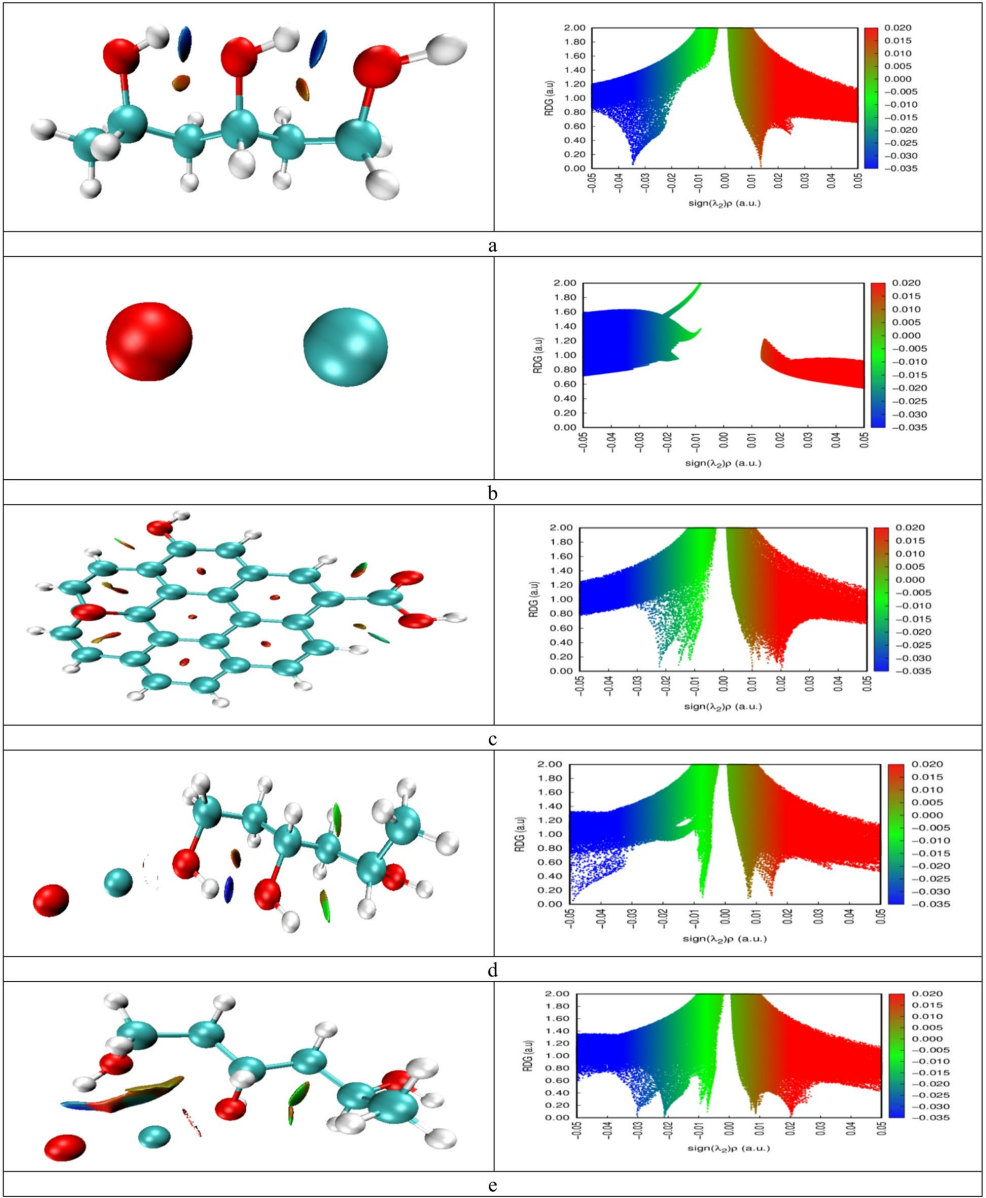

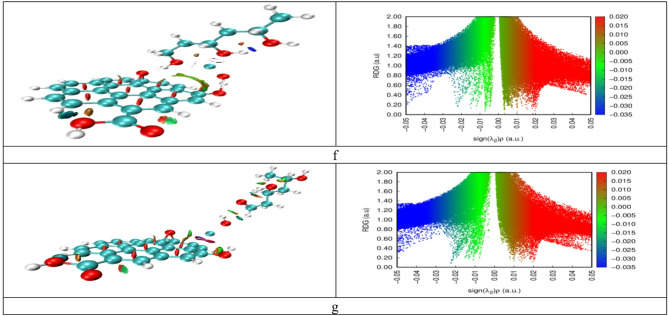
NCI isosurfaces and RDG plots for: (**a**) PVA, (**b**) CuO, (**c**) GO, (**d**) PVA-Cu/CuO, (**e**) PVA-O/CuO, (**f**) PVA-Cu/CuO/GO, and (**g**) PVA-O/CuO/GO. The domains, which are distinguished by color, differentiate between strong attractive (blue), van der Waals (green), and repulsive (red) interactions.

In order to clarify the intermolecular interactions in the composites, Reduced Density Gradient (RDG) mapping was conducted in conjunction with Non-Covalent Interaction (NCI) studies. These techniques utilize gradient plots and color-coded isosurfaces to illustrate weak non-bonded interactions, including hydrogen bonding, van der Waals forces, and steric repulsions^51^. In the NCI-RDG plots (Fig. 9), red areas indicate a strong steric repulsion, green areas signify weaker dispersive forces (van der Waals), and blue areas denote strong attractive interactions (hydrogen bonds). A clear blue signal in the RDG spectrum confirms hydrogen bonding between a hydrogen atom and the oxygen atom of PVA, as indicated by the blue isosurface in the NCI plot (Fig. 9-a). The NCI for CuO by itself is quite low (refer to Fig. 9-b). GO exhibits prominent red isosurfaces between its aromatic rings (Fig. 9-c) due to distortions from π-π stacking, indicating steric repulsion. The O and Cu atoms are involved in the interaction between PVA and CuO. Figure 9-d illustrates notable NCI attractions for the Cu and O atoms of PVA, while Fig. 9-e indicates that the PVA-O/CuO example exhibits a weak van der Waals interaction within PVA, as denoted by the green and RDG green spikes. PVA-Cu/CuO/GO and PVA-O/CuO/GO show comparable behavior (Fig. 9f and g). Nonetheless, PVA-O/CuO/GO displays a greater NCI and a marginally stronger attraction, which is counterbalanced by a somewhat increased repulsion and weaker contacts. This can result in a higher degree of stabilization than is observed with PVA-Cu/CuO/GO.

### Calculated global reactivity descriptors

Table 3 presents the DFT/B3LYP/LanL2DZ calculated global reactivity descriptors for the investigated structures PVA, CuO, GO, and their nanocomposites. The ionization potential (I) values indicate the ease of electron removal. Among the studied systems, PVA-Cu/CuO/GO exhibits the lowest I = 2.395 eV, implying enhanced electron-donating ability, which can promote charge transfer during sensing processes. Conversely, PVA-O/CuO/GO shows the highest I = 5.747 eV among the composites, reflecting greater electronic stability. The electron affinity (A), a measure of the tendency to accept electrons, is significantly higher for PVA-Cu/CuO/GO (4.886 eV) and PVA-O/CuO/GO (4.927 eV) compared to pristine PVA (−1.078 eV), suggesting that incorporation of OCu and GO considerably enhances electron-accepting capability, an essential feature for interaction with electron-rich analytes. The electronic chemical potential (µ) values follow a similar trend, with PVA-O/CuO/GO recording the highest value (5.337 eV), indicating strong electron-attracting tendencies and better charge redistribution upon adsorption of gas or humidity molecules. The chemical hardness (η) values reveal that PVA-O/CuO/GO possess the lowest hardness (0.410 eV), corresponding to the highest absolute softness (S) values (2.441 eV⁻¹). This high softness implies increased chemical reactivity and polarizability, making these systems more responsive to external stimuli. Finally, the electrophilicity index (ω), which quantifies the stabilization energy upon acquiring additional electronic charge, is markedly enhanced in PVA-O/CuO/GO (34.762 eV). The higher ω values show that the composites comprising H–O-Cu and GO have a strong electrophilic nature, which strengthens interactions with nucleophilic species and increases their surface reactivity. As a result, the PVA-O/CuO/GO composite exhibits a synergistic increase in electrophilicity, chemical softness, and electron affinity, all of which strengthen the composite’s overall chemical reactivity and greatly improve its detection and discrimination capabilities in environmental monitoring applications.

**Table 3:** Calculated global reactivity descriptors for the studied structures, including Ionization Potential (I), Electronic Affinity (A), Electronic chemical potential (µ), Chemical hardness (η), Absolute softness (S), and Electrophilicity index (ω), derived from DFT calculations at the B3LYP/LanL2DZ level of theory.

Structure	I (eV)	A (eV)	µ (eV)	η (eV)	S (eV⁻¹)	ω (eV)
PVA	6.255	−1.078	2.589	3.667	0.273	0.914
CuO	7.074	3.282	5.178	1.896	0.527	7.070
GO	5.897	2.523	4.211	1.687	0.593	5.256
PVA-Cu/CuO	5.318	0.068	2.693	2.625	0.381	1.381
PVA-O/CuO	6.021	0.819	3.420	2.601	0.3849	2.249
PVA-Cu/CuO/GO	2.395	4.886	3.641	1.245	0.803	5.322
PVA-O/CuO/GO	5.747	4.927	5.337	0.410	2.441	34.762

### Interaction of PVA/CuO/GO with H_2_O and CO_2_

#### TDM and HOMO-LUMO energy gap

Table 4 present TDM and HOMO-LUMO energy gap (ΔE) for PVA-Cu/CuO/GO and PVA-O/CuO/GO nanocomposites interacting with H₂O and CO₂ molecules. H₂O and 2 H₂O exhibit a moderate TDM 2.462 and 1.279 Debye, while maintaining a wide energy gap (9.811 and 9.796 eV respectively. In contrast, CO₂ and 2CO₂ show zero dipole moment with a constant ΔE of 9.920 eV due to their linear and nonpolar nature. Upon adsorption of H₂O and 2 H₂O on PVA-Cu/CuO/GO, a pronounced increase in TDM is observed (up to 10.469 and 10.447 Debye), accompanied by a significant reduction in ΔE from insulating values to 2.240 eV for H₂O and further to 1.112 eV for 2 H₂O, indicating strong charge redistribution and enhanced electronic conductivity. A similar trend is found for CO₂ adsorption, where ΔE decreases to 2.128 eV and 1.563 eV for CO₂ and 2CO₂, respectively, suggesting enhanced optical properties and higher electronic conductivity.

The structure PVA-O/CuO/GO −2CO₂ has the highest TDM (11.599 Debye) and a small ΔE (2.446 eV) compared to PVA-Cu/CuO/GO-2 H₂O, indicating strong optical activity but slightly poorer electrical conductivity. As a result, when considering both high TDM (for strong optical transitions) and low ΔE (for enhanced electronic properties), PVA-Cu/CuO/GO-2 H₂O is the most promising material for optoelectronic performance. However, due to its maximum TDM, PVA-O/CuO/GO is superior for sensing applications.

**Table 4:** B3LYP/LanL2DZ calculated total dipole moment (TDM) in Debye and HOMO-LUMO energy gap (∆E) in eV for H₂O, CO₂, and their interaction with PVA-Cu/CuO/GO and PVA-O/CuO/GO nanocomposites.

Structural	TDM (Debye)	ΔE (eV)
H_2_ O	2.462	9.811
2 H_2_ O	1.279	9.796
CO_2_	0.000	9.920
2CO_2_	0.000	9.920
PVA-Cu/CuO/GO-H_2_ O	10.469	2.240
PVA-Cu/CuO/GO-2 H_2_ O	10.447	1.112
PVA-Cu/CuO/GO-CO_2_	9.389	2.128
PVA-Cu/CuO/GO-2CO_2_	11.508	1.563
PVA-O/CuO/GO-H_2_ O	5.768	1.311
PVA-O/CuO/GO-2 H_2_ O	6.606	2.414
PVA-O/CuO/GO-CO_2_	7.975	2.794
PVA-O/CuO/GO-2CO_2_	11.599	2.446

#### MESP for PVA/CuO/GO interact with H_2_O and CO_2_

**Fig. 10:**
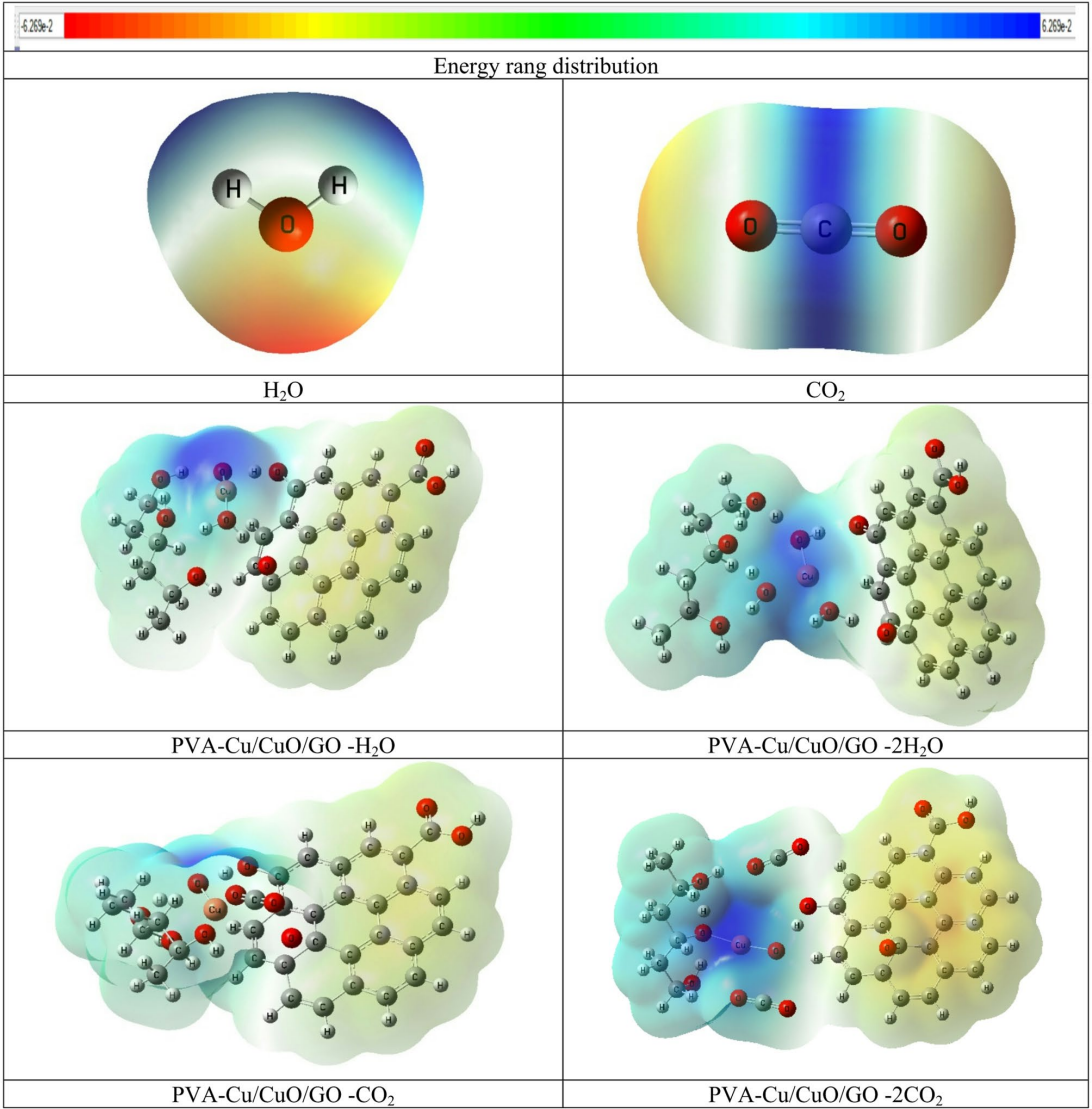

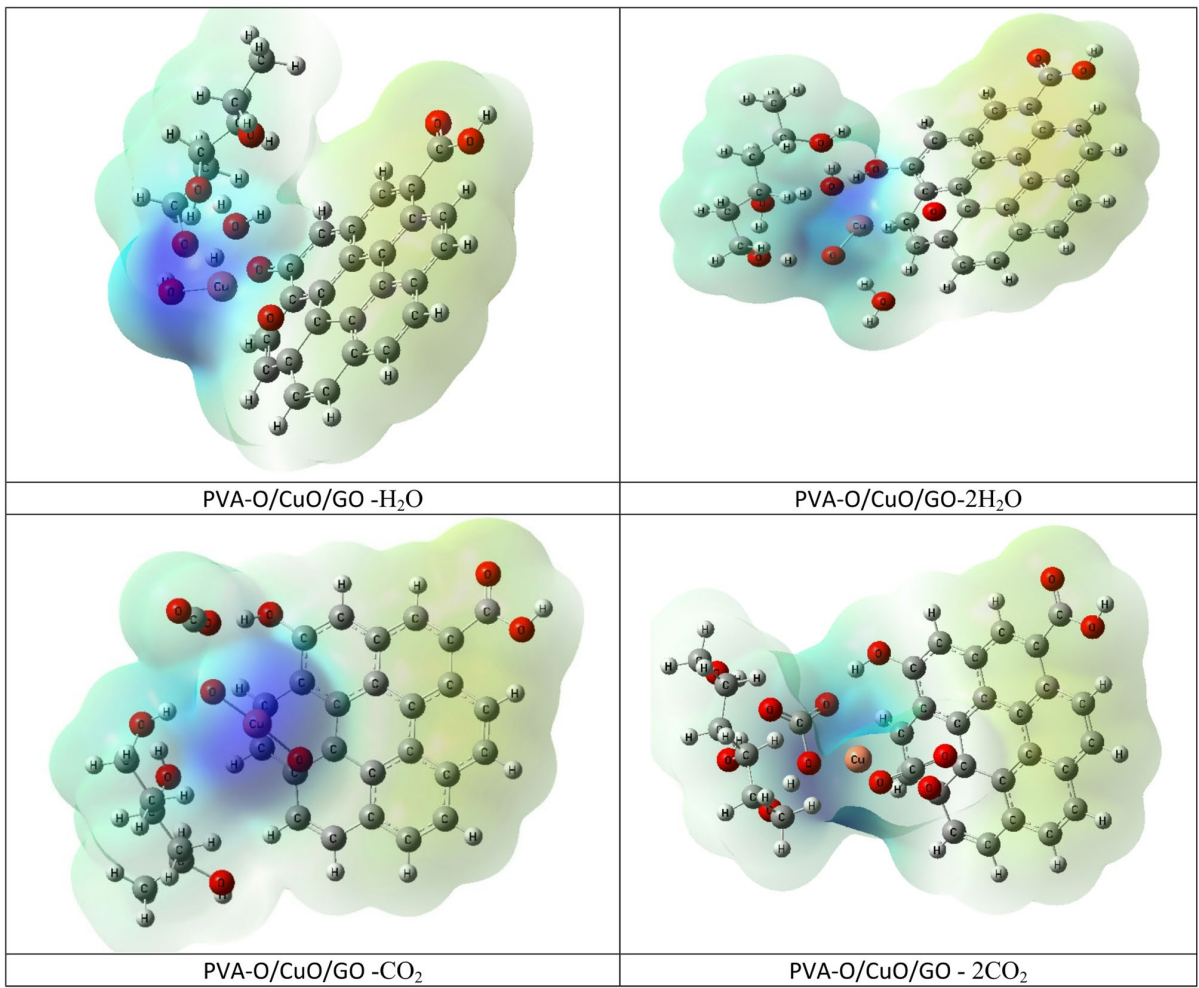
B3LYP/LanL2DZ calculated MESP for the interaction of PVA-Cu/CuO/GO and PVA-O/CuO/GO with H_2_O, 2 H_2_O, CO_2_ and 2CO_2_.

The molecular electrostatic potential (MESP) maps for PVA-Cu/CuO/GO and PVA-O/CuO/GO nanocomposites interacting with H₂O, 2 H₂O, CO₂, and 2CO₂ molecules are calculated and shown in Fig. 10. Yellow/orange outlines on these maps reflect intermediate potentials, blue parts show electron-deficient locations (positive potential), and red portions show electron-rich areas (negative potential). When these nanocomposites interact with H_2_O and CO₂, the measured color distributions show a considerable redistribution of charges, underscoring the composites strong chemical reactivity with these molecules. The PVA-O/CuO/GO and PVA-Cu/CuO/GO composites interacting with 2CO₂ (PVA-O/CuO/GO −2CO₂) exhibit the broadest high-potential zones and the highest polarization and activation among all examples. This is in line with its relatively narrow energy gap (2.446 eV) and maximum total dipole moment (TDM = 11.599 Debye), all of which point to improved charge transfer and reactivity. These characteristics show that the PVA-O/CuO/GO composite has considerable chemical reactivity to moisture and gases, with much stronger interactions seen when CO₂ is present.

#### Adsorption energy for PVA/CuO/GO interact with H_2_O and CO_2_

The adsorption energy is a crucial indicator of how strongly an atom or molecule interacts with a surface. The adsorption energies of the optimized structures were calculated in order to further examine the behavior of PVA/CuO/GO nanocomposites. The material surface contacts, reactivity, and attraction for other species are all revealed by the adsorption energy. As a result, determining the adsorption energy is crucial to comprehending the materials interaction potential and chemical performance^52^. It is the difference between the sum of the energies of the constituent parts before contact and the total energy of the combined system. The following formula^53^ was used to get the adsorption energy:

$${\mathrm{E}}_{{{\mathrm{Ads}}}} = {\text{ E}}_{{{\mathrm{System}}}} - {\text{ }}\left( {{\mathrm{E}}_{{{\mathrm{Adsorbent}}}} + ~{\mathrm{E}}_{{{\mathrm{Adsorbate}}}} } \right).$$

Table 5 summarizes the calculated total energies and adsorption energies for the PVA-Cu/CuO/GO and oxygen-rich PVA-O/Cu/GO nanocomposites interacting with H₂O and CO₂ molecules. The PVA-Cu/CuO/GO system exhibits a total energy of −54278.906 eV, which decreases to −56358.510 eV and − 58437.776 eV upon adsorption of one and two H₂O molecules, respectively, indicating enhanced structural stabilization induced by water adsorption. This stabilization is accompanied by moderate negative adsorption energies of −0.272 eV (H₂O) and − 0.206 eV (2 H₂O), suggesting a favorable and spontaneous interaction dominated by weak physisorption, primarily governed by hydrogen bonding and van der Waals forces. Similarly, the interaction of CO₂ with PVA-Cu/CuO/GO yields adsorption energies of − 0.217 eV and − 0.283 eV for one and two CO₂ molecules, respectively, along with a reduction in total energy to − 59409.560 eV and − 64540.064 eV. These relatively low adsorption energies indicate that CO₂ adsorption occurs mainly through weak van der Waals interactions accompanied by limited charge transfer, which is advantageous for reversible gas sensing applications. In contrast, the oxygen-enriched PVA-O/Cu/GO system exhibits a markedly different adsorption behavior. The adsorption of H₂O leads to more negative adsorption energies of −0.358 eV and − 0.396 eV for one and two molecules, respectively, with a pronounced decrease in total energy from − 54261.845 eV to −56341.535 eV and − 58420.905 eV. Likewise, CO₂ adsorption on PVA-O/Cu/GO becomes significantly enhanced, with adsorption energies of −0.365 eV for one CO₂ molecule and − 0.406 eV for two CO₂ molecules. These increased adsorption strengths indicate the presence of strong physisorption with partial chemisorptive character, arising from the availability of oxygenated Cu active sites that promote stronger molecule surface interactions and increased charge transfer. Overall, the numerical trends in Table 5 clearly demonstrate that raising the oxygen content significantly improves the PVA-O/Cu/GO nanocomposite adsorption affinity for both H_2_O and CO₂ molecules. This stronger interaction is expected to induce significant changes in the electronic structure, highlighting the PVA-O/Cu/GO system as a highly reactive and adaptable material for applications involving controlled interactions with these molecules.

**Table 5:** Calculated total energy and adsorption energy for the studied structures of PVA/CuO/GO and its interaction with H_2_O and CO_2_.

Structure	Total Energy (eV)	Adsorption Energy (eV)
H_2_ O	−2079.332	
2 H_2_ O	−4158.664	
CO_2_	−5130.437	
2CO_2_	−10260.875	
PVA-Cu/CuO/GO	−54278.906	
PVA-O/Cu/GO	−54261.845	
PVA-Cu/CuO/GO-H_2_ O	−56358.51	−0.272
PVA-Cu/CuO/GO-2 H_2_ O	−58437.776	−0.206
PVA-Cu/CuO/GO-CO_2_	−59409.56	−0.217
PVA-Cu/CuO/GO-2CO_2_	−64540.064	−0.283
PVA-O/Cu/GO-H_2_ O	−56341.535	−0.358
PVA-O/Cu/GO-2 H_2_ O	−58420.905	−0.396
PVA-O/Cu/GO-CO_2_	−59392.647	−0.365
PVA-O/Cu/GO-2CO_2_	−64523.126	−0.406

Similar adsorption-induced modifications in the electronic structure have been reported in recent first-principles investigations of gas-sensing nanomaterials, where adsorption of CO₂ and H₂ molecules generated significant redistribution of density of states near the Fermi level and enhanced charge-transfer behavior. These studies demonstrated that orbital hybridization between adsorbates and active surface sites plays a critical role in tuning sensing sensitivity and electronic conductivity, consistent with the present observations for the PVA/CuO/GO nanocomposites^31^.

These computational findings reveal the considerable sensing capability of the PVA/CuO/GO nanocomposites, as shown by the calculated changes in electronic properties and charge distribution upon analyte interaction. To supplement these theoretical results, potential experimental verification approaches like electrical measurements of conductivity/resistance variations or optical sensing methods might be utilized. Although the current research emphasizes thorough computational analyses, experimental studies are scheduled soon to verify and enhance the anticipated sensing capabilities.

## Conclusion

This study provided a comprehensive DFT investigation of PVA/CuO/GO hybrid nanocomposites and demonstrates that the synergistic incorporation of CuO and GO effectively tailors the electronic structure, interfacial interactions, and adsorption behavior of the PVA matrix. The calculated results confirm the formation of stable hybrid architectures governed by coordination interactions, hydrogen bonding, and dispersive forces, as validated by QTAIM and NCI-RDG analyses.

A pronounced enhancement in the electronic characteristics was achieved upon nanocomposite formation. The energy gap decreased dramatically from 7.334 eV for pristine PVA to 1.415 eV for PVA–Cu/CuO/GO and further to 0.819 eV for the oxygen-mediated PVA–O/CuO/GO configuration, indicating a transition from insulating to highly semiconducting behavior. Simultaneously, the total dipole moment increased from 7.148 Debye for pure PVA to 11.621 Debye for PVA–O/CuO/GO, reflecting strong charge polarization and enhanced electronic responsiveness. DOS calculations further confirmed the emergence of interfacial electronic states near the Fermi level due to orbital hybridization among Cu 3 d, O 2p, and C 2p orbitals, which promotes charge delocalization and facilitates electron transport across the hybrid interface. The calculated global reactivity descriptors revealed a substantial increase in chemical activity after incorporation of CuO and GO. In particular, PVA–O/CuO/GO exhibited a very low chemical hardness (η = 0.410 eV), high softness (S = 2.441 eV⁻¹), and remarkably high electrophilicity index (ω = 34.762 eV), demonstrating its strong polarizability and enhanced affinity toward external adsorbates. QTAIM analysis confirmed partially covalent Cu–O coordination interactions with electron density ρ(r) = 0.085 a.u. and negative total energy density H(r) = − 0.015 a.u., while NCI-RDG analysis revealed extensive hydrogen-bonding and van der Waals stabilization throughout the hybrid framework. Adsorption investigations demonstrated that the developed nanocomposites possess strong sensitivity toward H₂O and CO₂ molecules. Adsorption induced significant modulation of both dipole moment and energy gap, particularly for PVA–Cu/CuO/GO–2 H₂O (TDM = 10.447 Debye, ΔE = 1.112 eV) and PVA–O/CuO/GO–2CO₂ (TDM = 11.599 Debye, ΔE = 2.446 eV). The calculated adsorption energies ranged from − 0.206 to − 0.406 eV, indicating favorable spontaneous physisorption with partial charge transfer and good reversibility, which are desirable characteristics for sensing applications. Notably, the oxygen-rich PVA–O/CuO/GO system exhibited the strongest adsorption affinity toward both H₂O and CO₂, confirming the critical role of oxygen-active sites in enhancing analyte interaction and interfacial polarization.

Overall, the calculations established that cooperative integration of CuO and GO provides an efficient route for engineering the electronic and adsorption properties of PVA-based nanocomposites. The obtained theoretical insights reveal strong correlations between interfacial topology, charge redistribution, and sensing-related electronic descriptors, highlighting the PVA/CuO/GO system as a promising framework for the rational design of advanced multifunctional materials for sensing applications, warranting future experimental validation of their electronic and transport properties for future studies to validate and extend the predicted sensing performance.

## Data Availability

The data supporting the findings of this study can be obtained from the corresponding author upon request, subject to reasonable conditions.
